# Spatial distribution and risk factors for human cysticercosis in Colombia

**DOI:** 10.1186/s13071-021-05092-8

**Published:** 2021-11-27

**Authors:** Erika Galipó, Matthew A. Dixon, Claudio Fronterrè, Zulma M. Cucunubá, Maria-Gloria Basáñez, Kim Stevens, Astrid Carolina Flórez Sánchez, Martin Walker

**Affiliations:** 1 Department of Epidemiological Sciences, Animal and Plant Health Agency, New Haw, Addlestone, Surrey UK; 2 Department of Pathobiology and Population Sciences and London Centre for Neglected Tropical Disease Research, Royal Veterinary College, Hatfield, UK; 3 Department of Infectious Disease Epidemiology and London Centre for Neglected Tropical Disease Research, School of Public Health, Imperial College London, London, UK; 4 Medical Research Centre for Global Infectious Disease Analysis, School of Public Health, Imperial College London, London, UK; 5 Schistosomiasis Control Initiative (SCI) Foundation, Edinburgh House, 170 Kennington Lane, Lambeth, London, SE11 5DP UK; 6 Centre for Health Informatics, Computing and Statistics, Lancaster University, Lancaster, UK; 7 Grupo de Parasitología, Instituto Nacional de Salud, Bogotá, Colombia; 8 Present Address: Departamento de Epidemiología Clínica, Pontificia Universidad Javeriana, Bogotá, Colombia

**Keywords:** *Taenia solium*, Cysticercosis, Risk factors, Spatial analysis, Geostatistics, Colombia

## Abstract

**Background:**

Cysticercosis is a zoonotic neglected tropical disease (NTD) that affects humans and pigs following the ingestion of *Taenia solium* eggs. Human cysticercosis poses a substantial public health burden in endemic countries. The World Health Organization (WHO) aims to target high-endemicity settings with enhanced interventions in 17 countries by 2030. Between 2008 and 2010, Colombia undertook a national baseline serosurvey of unprecedented scale, which led to an estimated seroprevalence of *T. solium* cysticercus antibodies among the general population of 8.6%. Here, we use contemporary geostatistical approaches to analyse this unique dataset with the aim of understanding the spatial distribution and risk factors associated with human cysticercosis in Colombia to inform how best to target intervention strategies.

**Methods:**

We used a geostatistical model to estimate individual and household risk factors associated with seropositivity to *T. solium* cysticercus antibodies from 29,253 people from 133 municipalities in Colombia. We used both independent and spatially structured random effects at neighbourhood/village and municipality levels to account for potential clustering of exposure to *T. solium*. We present estimates of the distribution and residual correlation of seropositivity at the municipality level.

**Results:**

High seroprevalence was identified in municipalities located in the north and south of Colombia, with spatial correlation in seropositivity estimated up to approximately 140 km. Statistically significant risk factors associated with seropositivity to *T. solium* cysticercus were related to age, sex, educational level, socioeconomic status, use of rainwater, consumption of partially cooked/raw pork meat and possession of dogs.

**Conclusions:**

In Colombia, the distribution of human cysticercosis is influenced by socioeconomic considerations, education and environmental factors related to the spread of *T. solium* eggs. This information can be used to tailor national intervention strategies, such as targeting spatial hotspots and more highly exposed groups, including displaced people and women. Large-scale seroprevalence surveys accompanied by geospatial mapping are an essential step towards reaching the WHO’s 2021‒2030 NTD roadmap targets.

**Graphical Abstract:**

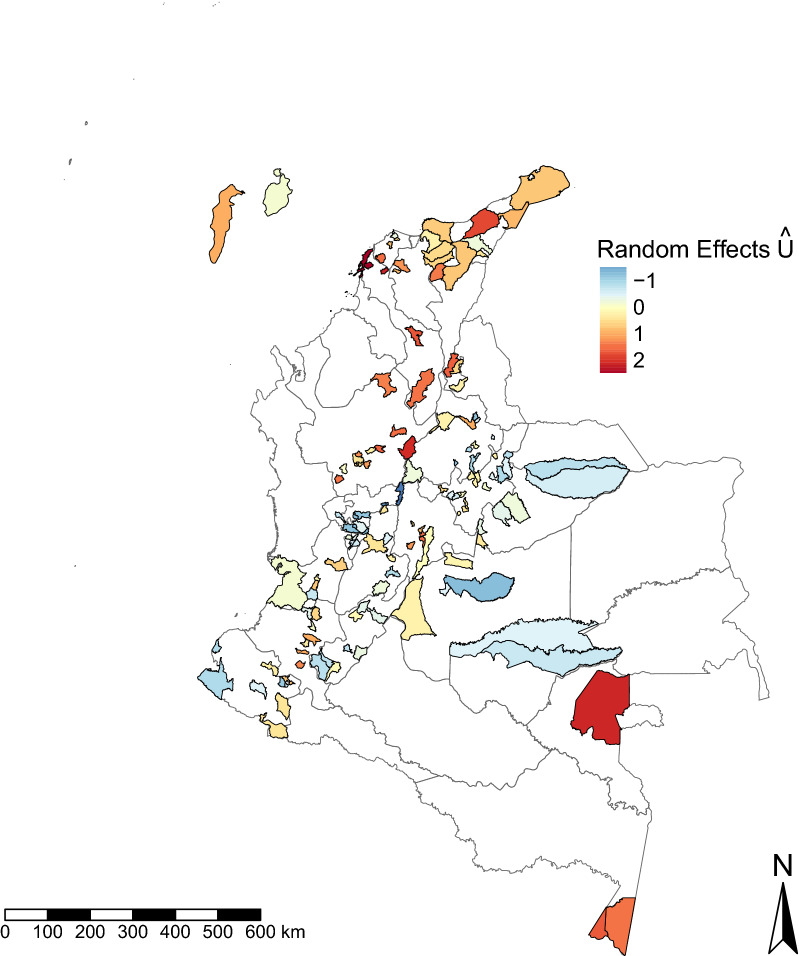

**Supplementary Information:**

The online version contains supplementary material available at 10.1186/s13071-021-05092-8.

## Background

The zoonotic tapeworm, *Taenia solium*, is responsible for taeniasis/cysticercosis which is included in the World Health Organization’s (WHO’s) list of prioritised neglected tropical diseases (NTDs) [1]. Humans are the definitive hosts of *T. solium* and harbour the adult tapeworm in their bowel. Pigs are intermediate hosts, infected by larval cysts (cysticerci) following ingestion of parasite eggs and proglottids [2] in human faeces. Eggs hatch in the pig’s digestive system, and the released oncospheres first penetrate the intestinal wall, entering the bloodstream, and then become encysted in striated muscle, brain, liver and subcutaneous and other tissues. Porcine cysticercosis is often asymptomatic [2, 3], although cysts in pig brain tissue can cause neurocysticercosis (NCC) and epileptic seizures [4].

Humans contract taeniasis following consumption of tissue cysts in poorly cooked pork meat. Taeniasis is usually asymptomatic, but mild symptoms, including abdominal pain, distension, diarrhoea and nausea, may appear [2]. Humans can also be infected with *T. solium* eggs, typically from ingestion of food contaminated with human faecal material [5] or food washed with contaminated water [6]. Internal auto-infestation following regurgitation of proglottids in the stomach has also been suggested as an additional route of infection [2, 5, 7]. Infection with *T. solium* eggs causes cysticercosis which manifests most severely when cysts migrate to the central nervous system, resulting in NCC [2]. Morbidity from NCC associated with seizures, epilepsy and other neurological sequelae is driven by the number and location of cysts or following the degeneration of viable cysts [8].

Taeniasis/cysticercosis is widely endemic globally. *Taenia solium* cysticercosis antibody seroprevalence, indicative of exposure, ranges from 1.8 to 31.2% in Latin America, from 12.6 to 19.2% in Asia and from 7.7 to 34.5% in Africa (as measured using an enzyme-linked Immunoelectrotransfer blot [EITB] assay) [9], which highlights substantial variation in exposure to *T. solium* eggs across settings. NCC is responsible for the predominant disease burden associated with *T. solium* infection, accounting for approximately 30% of epilepsy cases in endemic countries and 3% globally [10]. In addition, this zoonosis impacts the pork meat market, with small producers experiencing economic losses due to the reduction in value of infected pork meat [4] and a market shift towards home slaughtering and selling [11].

In Colombia, taeniasis/cysticercosis poses a substantial public health problem [12], with an estimated life-time prevalence of epilepsy of 20.9 per 1000 individuals and a prevalence of neurocysticercosis (by computed tomography scan) of 13.9% [13]. The country-wide prevalence of *T. solium* cysticercus antibodies was estimated at 8.6% from a national serosurvey of more than 29,000 people conducted between 2008 and 2010 [14]. Despite the unprecedented scale of this epidemiological survey—and the development by the Pan American Health Organization in 2015 of a formal plan of surveillance and control in Colombia [12]—there has been little implementation of systematic surveillance or intervention activities. Consequently, the epidemiology of *T. solium* in Colombia is unlikely to have changed substantively during the past decade since these data were generated. Thus, the dataset remains the most comprehensive and relevant country-wide cross-sectional ‘snapshot’ of *T. solium* epidemiology anywhere across the globe and a unique information resource.

Here, we analyse this dataset using a contemporary geostatistical approach to understand the spatial distribution of *T. solium* cysticercus seropositivity in Colombia, as well as individual and household risk factors associated with exposure to the parasite. This work extends the original analysis of these data [14] by integrating the effects of individual covariates and spatial clustering at multiple hierarchical levels within a single statistical framework. We present maps of the spatial distribution of *T. solium* cysticercus seropositivity in Colombia, estimates of spatial correlation and demographic, socioeconomic, behavioural and other risk factors associated with exposure to this zoonotic NTD.

## Methods

### Study design

The data were collected by the Colombian National Health Institute (Instituto National de Salud) between 2008 and 2010 with the aim of estimating *T. solium* human cysticercosis antibody seroprevalence and associated risk factors. Details of the original data collection can be found in [14]. Briefly, individuals aged from 2 to 64 years, from 23 departments and Bogotá district, living in 133 municipalities with > 5000 inhabitants and a health centre were eligible for inclusion. The small proportion of total municipalities sampled (133/1122) was due to logistical and financial constraints. A three-stage cluster random sampling approach was used, covering 23 out of Colombia’s 32 departments (first administrative level unit) and Bogotá district (Additional file 1: Figure S1). The municipality constituted the primary sample unit (PSU) and was stratified according to level of urbanization, rural and urban population composition and the Unsatisfied Basic Needs Index (Indice de Necesidades Básicas Insatisfechas) [15]. Within each stratum, the secondary sample unit (SSU) was defined as a neighbourhood (urban) or village (rural) with > 10 households and selected by random sampling. Finally, 10 households in each SSU were randomly selected, and one person belonging to each household (between the age of 2 and 64 years) was selected at random from those present at the interview. Following informed consent, finger-prick blood samples were obtained from 29,360 participants, and each sample was assessed for the presence of circulating *T. solium* cysticercus antibodies at the National Health Institute Reference Laboratory (Laboratorio de Parasitología del Instituto Nacional de Salud) by enzyme-linked immunosorbent assay (ELISA), with a reported sensitivity of 100% and specificity of 97.5% [16]. Participants also completed a questionnaire on sociodemographic information, hygiene habits, health conditions, food consumption habits, living conditions and animal ownership and management. The questionnaire was developed by the research team in Colombia, with input from experts on cysticercosis. It was first tested in a pilot survey carried out in 216 homes in the municipality of Caqueza (Department of Cundinamarca), from 28 August to 2 September 2008 and adjusted accordingly. Teams in the field were trained on the use of the questionnaire before it was applied on the whole sample. Details on the cleaning and coding of this dataset can be found in Additional file 1: Text S1.

### Model-building and analysis of residual spatial correlation

Before performing the geospatial analysis, an initial exploratory analysis was undertaken (using R version 4.0.5 [17]). Given the clustered nature of the data, a hierarchical univariate mixed-effects logistic regression model was fitted to test the association between each explanatory variable (covariate) and human seropositivity to *T. solium* cysticerci, with each model including two independent random effects terms to capture correlation at the municipality and neighbourhood/village (depending on urban or rural location) levels. Explanatory variables with a *P-*value ≤ 0.25 (a conservative cut-off to avoid missing potentially important variables), derived from a likelihood ratio test, were retained in the subsequent hierarchical multivariable mixed-effects logistic regression model.

The generic structure of all models is given by:

1 $$\begin{array}{*{20}c} {\varvec{Y}~\sim ~Bern\left( \user2{\mu } \right),~} \\ {logit\left( \user2{\mu } \right) = \mathbf{\beta X~ + Z~ + U,} } \\ {\varvec{Z}~\sim ~N\left( {0,~\tau } \right),} \\ {\varvec{U}~\sim ~N\left( {0,~\sigma } \right),~} \\ \end{array}$$

where ${\varvec{Y}}$ is a binary vector of observations indicating whether an individual tested positive for *T. solium* cysticercus antibodies, assuming a Bernoulli distribution; ${\varvec{\mu}}$ is a vector of probabilities for testing positive; ${\varvec{\beta}}$ is a vector of regression coefficients, and $\mathbf{X}$ is the design matrix of explanatory variables; ${\varvec{U}}$ and ${\varvec{Z}}$ are vectors of independent and normally distributed random effects terms associated with municipalities and neighbourhoods/villages, respectively; and $\sigma$ and $\tau$ are the standard deviations of the respective random effects terms (indicative of the degree of variability at each hierarchical level). From the final fitted models, adjusted odds ratios (ORs), 95% confidence intervals (95% CIs) and *P*-values were obtained for each risk factor. All notations/parameters are summarised in Additional file 1: Table S1. A sub-analysis on risk factors in those individuals owning pigs (*n* = 3154) was also conducted (methodological details are given in Additional file 1: Text S1).

Following fitting of the multivariable mixed-effects model, a variogram analysis was performed to assess the presence of residual spatial correlation [17]. Since the geographical coordinates were available only for the municipalities and not for the neighbourhoods/villages, the empirical variogram was computed only on $\widehat{{\varvec{U}}}$, the estimated random effects at the municipality level. A Monte Carlo test for the null hypothesis of spatial independence was performed based on 10,000 random permutations of $\widehat{{\varvec{U}}}$ amongst the sampled municipalities. The variograms computed on the permuted random effects represent the sampling distribution of the estimated variogram in the absence of spatial correlation. If the empirical variogram ordinates fall outside of the 95% CI obtained from the Monte Carlo test, then there is some evidence of spatial correlation at municipality level.

### Incorporating spatial structure

In the presence of spatial correlation, the independent random effects at the municipality level, ${\varvec{U}}$, were replaced with a set of spatially structured random effects, ${\varvec{S}}({\varvec{x}})$, where ${\varvec{x}}$ is a vector with the centroids of the sampled municipalities. ${\varvec{S}}({\varvec{x}})$ is a spatial Gaussian process with variance ${\sigma }^{2}$ and correlation function$\rho \left(\mu \right)=\mathrm{exp}\left(-\frac{\mu }{\varphi }\right)$, where $\mu$ is the distance between a pair of municipality centroids and $\varphi$ is a parameter that controls the rate at which the spatial correlation decays with increasing distance. Conditional on these spatially structured random effects, the observations can still be considered as independent Bernoulli random variables [18]. The spatially structured model was fitted using the integrated nested Laplace approximation (INLA) and stochastic partial differential equation (SPDE) approaches [19, 20] which implement approximate Bayesian inference in a computationally less intensive manner to alternative Markov chain Monte Carlo (MCMC) approaches. A flat Gaussian prior with mean and precision equal to zero was assigned to the model intercept term; other fixed effects were assigned independent vague Gaussian priors with mean zero and precision equal to 0.001. For the precision of the independent neighbourhood/village random effects, $1/\tau$, a vague Gamma prior was used, and for the parameters ${\sigma }^{2}$ and $\varphi$ of the spatially-structured random effects, we adopted penalised complexity priors [21]. Adjusted ORs and 95% credible intervals (95% CrIs) were obtained for each risk factor from the final fitted model.

## Results

### Study population and seroprevalence distribution

**Fig. 1:**
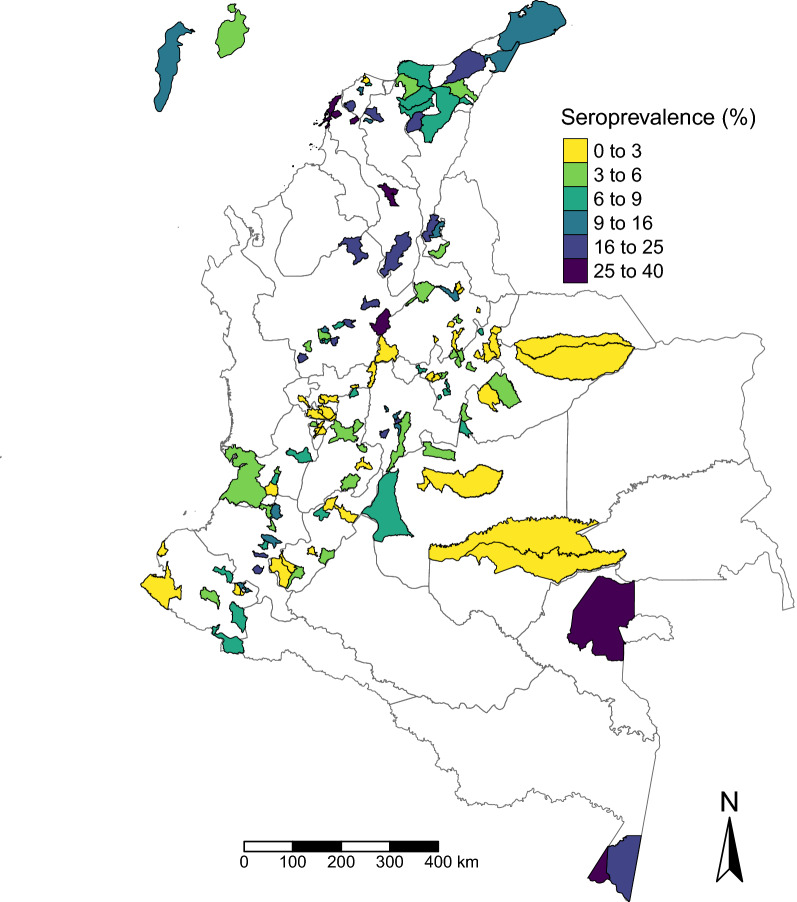
Seroprevalence of cysticercosis in Colombia, 2008–2010. Seroprevalence of *Taenia solium* cysticercus antibodies in 133 municipalities in Colombia. Departments are outlined in pale grey lines and sampled municipalities are shown in solid colours

Of the 29,360 observations, 29,253 (99.6%) observations were kept for analysis, with 107 removed due to missing covariate values. Participants were mostly located in urban areas (77.9%), mostly aged 21‒50 years (64.4%) and mostly women (68.5%); the main occupational activity was housewife/houseman (44.5%). Socioeconomic stratum 1 (lowest of 4 socioeconomic strata, excluding displaced people) was the most frequently represented socioeconomic stratum (49.5%), and participants most frequently had a partial or complete secondary school educational level (45.8%) (Table 1). The mean seroprevalence of *T. solium* cysticercus antibodies was 9.6%, ranging from 0.5% in the Department of Caldas to 38.7% in the Department of Vaupés (Additional file 1: Table S2). Municipalities with the highest seroprevalence were located in the north and south of Colombia (Fig. 1), while municipalities with lower seroprevalence were concentrated in the central part of the country.

**Table 1:** Total number of respondents for each covariate level and total number positive for circulating *Taenia solium* cysticercus antibodies

Covariate	Level	Number (%) of respondents	Total number (%; 95% CI) of positive respondents
Sociodemographic characteristics			
Sex	Male	9227 (31.54)	809 (8.77; 8.20–9.36)
	Female	20,026 (68.46)	1967 (9.82; 9.41–10.20)
Age groups (years)	2–10	972 (3.32)	76 (7.82; 6.21–9.69)
	11–20	3583 (12.25)	337 (9.41; 8.47–10.4)
	21–30	6558 (22.42)	641 (9.78; 9.07–10.50)
	31–40	6359 (21.74)	617 (9.70; 8.99–10.50)
	41–50	5927 (20.26)	578 (9.76; 9.01–10.50)
	51–60	4398 (15.03)	409 (9.30; 8.46–10.20)
	61–64	1456 (4.98)	117 (8.04; 6.70–9.56)
Residence	Rural	6455 (22.07)	764 (11.80; 11.00–12.60)
	Urban	22,798 (77.93)	2011 (8.83; 8.46–9.20)
Education level	Education higher than secondary	3862 (13.20)	296 (7.67; 6.85–8.55)
	Partial/complete secondary education	13,405 (45.82)	1190 (8.88; 8.40–9.37)
	Partial/complete primary education	10,615 (36.29)	1113 (10.5; 9.91–11.10)
	No education	1371 (4.69)	176 (12.8; 11.10–14.70)
Occupation	Other occupations	844 (6.17)	125 (6.93; 5.80–8.20)
	Self-employed	4340 (14.84)	325 (7.49; 6.72–8.31)
	Employee	4367 (14.93)	374 (8.56; 7.75–9.43)
	Farm coordinator	135 (0.46)	12 (8.89; 4.68–15.00)
	Farm labourer	720 (2.46)	65 (9.03; 7.04–11.40)
	Student	3538 (12.09)	322 (9.10; 8.18–10.10)
	Housewife/houseman	13,005 (44.46)	1313 (10.1; 9.58–10.6)
	Businessman	499 (1.71)	53 (10.6; 8.06–13.7)
	Farm owner	1805 (2.89)	186 (22.00; 19.30–25.00)
Socioeconomic status (stratum)	≥ 4	581 (1.99)	26 (4.48; 2.94–6.49)
	3	3743 (12.80)	176 (4.70; 4.05–5.43)
	2	10,166 (34.75)	706 (6.95; 6.46–7.46)
	1	14,465 (49.45)	1715 (11.90; 11.30–12.40)
	Displaced people	298 (1.02)	152 (51.00; 45.20–56.80)
Eating habits			
Pork consumption & cooking level	No consumption	3328 (11.38)	416 (12.50; 11.40–13.70)
	Well cooked; < once per month	12,744 (43.56)	1203 (9.44; 8.94–9.96)
	Well cooked; once per month	5789 (19.79)	561 (9.69; 8.94–10.5)
	Well cooked; once per week	3419 (11.69)	293 (8.57; 7.65–9.56)
	Well cooked; > once per week	1549 (5.29)	127 (8.20; 6.88–9.68)
	Partially cooked/raw; < once per month	1320 (4.51)	96 (7.27; 5.93–8.81)
	Partially cooked/raw; once per month	436 (1.49)	32 (7.34; 5.07–10.2)
	Partially cooked/raw; once per week	410 (1.40)	26 (6.33; 4.17–9.13)
	Partially cooked/raw; > once per week	265 (0.91)	21 (7.92; 4.97–11.9)
Water source	Well/ cistern	2738 (9.36)	190 (6.94; 6.02–7.96)
	Aqueduct	19,485 (66.61)	1401 (7.19; 6.83–7.56)
	Waterway	4904 (16.77)	564 (11.50; 10.60–12.5)
	Other sources	574 (1.96)	132 (23.00; 19.60–26.70)
	Rain water	1554 (5.31)	488 (31.40; 29.10–33.70)
Hygiene practices			
Washing vegetables	No consumption	82 (0.28)	8 (9.76; 4.31–18.30)
	Always	7304 (24.97)	588 (8.05; 7.43–8.69)
	Occasionally	10,664 (36.46)	1461 (13.7; 13.00–14.30)
	Never	11,166 (38.17)	718 (6.43; 5.98–6.90)
Washing hands before a meal	Always	12,925 (44.18)	1171 (9.06; 8.58–9.57)
	Occasionally	14,950 (51.11)	1489 (9.96; 9.48–10.40)
	Never	1376 (4.70)	115 (8.36; 6.95–9.95)
Elimination of excreta	Sanitary conditions	20,401 (69.74)	1526 (7.48; 7.12–7.85)
	In waterway	543 (1.86)	50 (9.21; 6.91–12.00)
	Latrine with well	6403 (21.89)	862 (12.9; 12.1–13.7)
	Latrine without well	519 (1.78)	67 (12.9; 10.1–16.1)
	Open field	1084 (3.71)	270 (24.9; 22.3–27.5)
Owning animals			
Cattle	Not owning	27,947 (95.54)	2641 (9.45; 9.11–9.80)
	Owning	1301 (4.45)	134 (10.3; 8.74–12.1)
Cats	Not owning	23,590 (80.64)	2208 (9.36; 8.99–9.74)
	Owning	5670 (19.38)	567 (10.0; 9.26–10.8)
Dogs	Not owning	16,194 (55.36)	1349 (8.33; 7.91–8.77)
	Owning	13,083 (44.72)	1426 (10.9; 10.4–11.5)
Birds	Not owning	20,213 (69.10)	1799 (8.90; 8.51–9.30)
	Owning	9037 (30.89)	976 (10.80; 10.20–11.50)
Pigs	Not owing	26,105 (89.24)	2386 (9.14; 8.80–9.50)
	Owning ≤ 10 pigs	2705 (9.25)	349 (12.9; 11.70–14.30)
	Owning > 10 pigs	460 (1.57)	40 (8.70; 6.28–11.7)

*CI* Confidence interval

### Risk factors for human seropositivity without spatial structure

From the univariate mixed-effects logistic regression model with two random effects, food consumption in streets, washing hands after toilet usage and owning animals other than dogs and pigs (cattle, cats, birds) were excluded from further (multivariate) analysis, having a *P-*value > 0.25 (Additional file 1: Table S3). Consequently, 16 explanatory variables were included in the multivariable mixed-effect logistic regression with two random effects. Increasing age (as age categories), being female, owning dogs and using rainwater as a water source were significantly associated with increased odds of being seropositive for *T. solium* cysticercus antibodies; increasing education level, socioeconomic status and consuming partially cooked/raw pork meat once per week were significantly associated with decreased odds of being seropositive (Additional file 1: Table S4). Risk factor analysis results from the sub-analysis of those owning pigs (*n* = 3154) are reported in Additional file 1: Text S2 and Additional file 1: Table S5.

### Geographical variation in random effects and spatial correlation

**Fig. 2:**
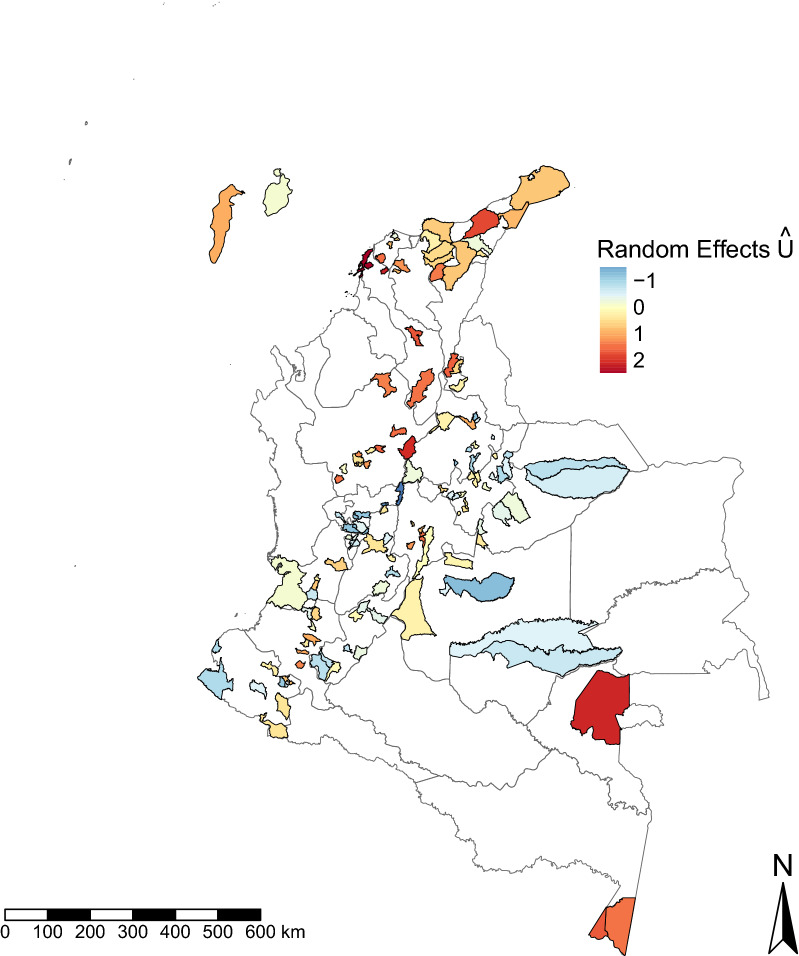
Residual variation in* Taenia solium* cysticercus seroprevalence at the municipality level across Colombia. The map represents the residual variation in cysticercus seroprevalence at the municipality level that is not explained by the covariates in the non-spatial mixed-effects model

**Fig. 3:**
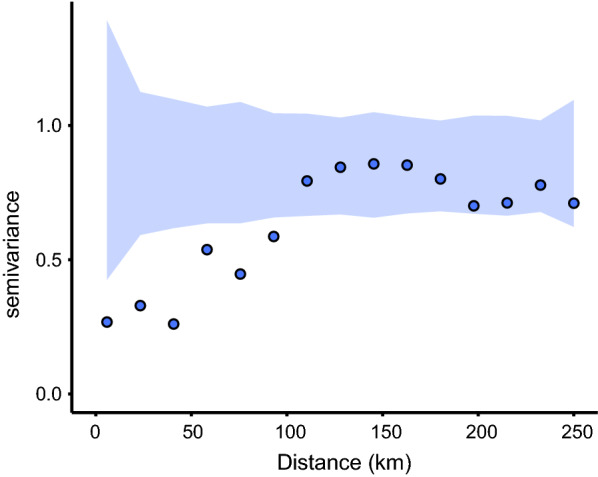
Estimated variogram for the mixed-effects model residuals at the municipality level (blue dots) across Colombia, including 95% confidence intervals obtained from a permutation test under the null hypothesis that there is no spatial correlation (blue-shaded area). The blue dots fall outside of the confidence bands up to approximately 120–140 km of separation, indicating spatial correlation up to this distance (confirmed by the geostatistical model)

Figure 2 shows a map of the residual variation in the seroprevalence of *T. solium* cysticercus antibodies at the municipality level that is unexplained by the covariates in the non-spatial mixed-effects model. Figure 3 shows a variogram analysis carried out on the municipalities’ estimated random effects. The empirical variogram falls partially outside of the 95% confidence bands, suggesting the presence of spatial correlation in seroprevalence at the municipality level (unexplained by the covariates) up to approximately 120–140 km; further than this distance, the variation between two spatial points starts to plateau This estimate was determined more precisely from the fitted geostatistical model (see below) to a value of 139 km.

### Geostatistical model

The geostatistical model estimated a strong spatial correlation at the municipality level of up to 139 km. The ORs and 95% CrIs associated with each covariate included in the final multivariable model (which accounts for spatial correlation at the municipality level) are given in Table 2. Notably, the odds of testing positive for *T. solium* cysticercus antibodies was 1.29-fold (95% CrI = 1.15–1.46) greater for females than for males, and the odds of testing positive generally increased with age. For example, adults aged between 21 and 60 years were approximately twofold more likely to test positive than children in the age range 2–10 years. Lower educational levels were significantly associated with increased odds of seropositivity, with the highest estimated odds associated with no formal education. Displaced people had 2.20-fold (95% CrI = 1.15–4.28) higher odds of being seropositive than people in the highest socioeconomic stratum; there was no significant difference among other socioeconomic strata. The use of rainwater as a water source was associated with 1.6-fold (95% CrI = 1.21–2.13) higher odds of being positive compared to the use of a well or cistern, and dog owners were at significantly increased odds of testing positive (OR = 1.19, 95% CrI = 1.08–1.31) than non-owners. Consumption of partially cooked/raw pork meat once per week was associated with a significantly decreased odds of testing positive (OR: 0.59, 95% CrI: 0.36 – 0.90) compared to no consumption. Place of residence, occupation, frequency of washing vegetables, excreta elimination and owning animals other than dogs (including pigs) were not significantly associated with testing positive for* T. solium* cysticercus antibodies.

**Table 2:** Geostatistical multivariable logistic regression model results: odds of testing positive for *Taenia solium* cysticercus antibodies

Covariate	Level	OR (95% CrI)
Sociodemographic characteristics		
Sex	Male	1
	Female	1.29 (1.15–1.46)*
Age group (years)	2–10	1
	11–20	1.83 (1.35–2.50)*
	21–30	1.96 (1.43–2.72)*
	31–40	1.96 (1.42–2.73)*
	41–50	2.12 (1.54–2.95)*
	51–60	2.00 (1.45–2.80)*
	61–64	1.68 (1.16–2.44)*
Residence	Rural	1
	Urban	0.84 (0.69–1.02)
Education level	Higher than secondary	1
	Partial/complete secondary	1.05 (0.90–1.23)
	Partial/complete primary	1.32 (1.11–1.57)*
	No education	1.34 (1.04–1.73)*
Occupation	Other occupations	1
	Self-employed	1.01 (0.80–1.29)
	Employee	1.07 (0.85–1.36)
	Farm coordinator	1.09 (0.52–2.11)
	Farm labourer	1.06 (0.73–1.52)
	Student	1.20 (0.91–1.58)
	Housewife/houseman	1.07 (0.86–1.36)
	Businessman	1.22 (0.83–1.79)
	Farm owner	1.20 (0.89–1.63)
Socioeconomic status	≥ 4	1
	3	0.93 (0.57–1.55)
	2	1 (0.63–1.64)
	1	1.12 (0.70–1.86)
	Displaced	2.20 (1.15–4.28)*
Eating habits		
Pork consumption	No consumption	1
	Well cooked; < once per month	0.92 (0.80–1.06)
	Well cooked; once per month	0.94 (0.80–1.11)
	Well cooked; once per week	0.85 (0.71–1.03)
	Well cooked; > once per week	0.98 (0.76–1.24)
	Partially cooked/raw; < once per month	0.85 (0.64–1.11)
	Partially cooked/raw; once per month	0.89 (0.58–1.33)
	Partially cooked/raw; once per week	0.59 (0.36–0.90)*
	Partially cooked/raw; > once per week	0.94 (0.55–1.22)
Water source	Well/cistern	1
	Aqueduct	1.06 (0.84–1.33)
	Waterway	1.12 (0.88–1.45)
	Other sources	1.11 (0.78–1.58)
	Rain water	1.60 (1.21–2.13)*
Hygiene practices		
Washing vegetables	No consumption	1
	Always	1.20 (0.56–2.92)
	Occasionally	1.28 (0.60–3.12)
	Never	1.17 (0.55–2.86)
Washing hands before a meal	Always	1
	Occasionally	1.04 (0.94–1.15)
	Never	0.90 (0.71–1.14)
Elimination of excreta	Sanitary conditions	1
	In waterway	0.84 (0.55–1.26)
	Latrine with well	1.06 (0.90–1.25)
	Latrine without well	1.16 (0.82–1.62)
	Open field	1.10 (0.85–1.42)
Owning animals		
Cattle	Not owning	1
	Owning	0.97 (0.76–1.22)
Cats	Not owning	1
	Owning	1.07 (0.95–1.20)
Dogs	Not owning	1
	Owning	1.19 (1.08–1.31)*
Birds	Not owning	1
	Owning	0.96 (0.86–1.07)
Pigs	Not owing	1
	Owning ≤ 10 pigs	1.10 (0.93–1.31)
	Owning > 10 pigs	0.83 (0.56–1.19)

* Crl* Credible interval,* OR* odds ratio

*Statistically significant

## Discussion

The 2008–2010 Colombian cysticercosis serosurvey generated unique and unprecedented information on exposure to *T. solium* cysticercosis at a national scale. The work presented here extends the original analysis of these data [14] by using contemporary geostatistical techniques to evaluate individual-level risk factors associated with seropositivity to *T. solium* cysticerci and, simultaneously, spatial clustering at a sub-national (municipality) scale. The results contribute important information on factors associated with exposure to *T. solium* cysticerci. They also indicate that similar large-scale epidemiological surveys will be needed if hyperendemic foci of transmission are to be identified and targeted for intensified interventions in 17 endemic countries, as per the WHO’s 2021–2030 NTD roadmap targets for taeniasis/cysticercosis [22].

Here, and in the original analysis of these data [14], women were more likely than men to be positive for *T. solium* cysticercus antibodies. This finding is consistent with the results of numerous other studies undertaken in Latin America [2, 9, 23–27]; by contrast, in other endemic regions, such as sub-Saharan Africa, being male is associated with an increased risk of exposure [28] and of antigen positivity [29, 30]. The mechanisms underlying these epidemiological patterns remain unclear. Different household roles associated with handling household-owned animals, food and water may be important, although many variables pertaining to these activities were accounted for in this analysis. Notwithstanding the underlying cause, women could be an important target for educational campaigns in Colombia, not just because of their apparent increased risk of exposure, but also because they are often being responsible for the majority of food handling and preparation activities, which would be all the more important if they were also tapeworm carriers.

The trend for increasing seropositivity with age is unsurprising given that *T. solium* cysticercus antibodies probably persist for several years. Seropositivity may thus be considered as an indicator of lifetime prior exposure. Praet et al. [31] explored age-dependent dynamics of *T. solium* cysticercus antibody positivity in more depth by fitting mathematical models to similar age–seroprevalence data collected in Ecuador. Their results suggested that higher antibody seroreversion rates occur following first exposure (representing the primary humoral response), followed by a lower seroreversion rate after the boosting effect of subsequent exposures (representing secondary humoral response), causing saturation in antibody seroprevalence with age. Hence, where transmission is relatively intense—and repeated exposures are common—one might expect to see similar saturating age–seroprevalence profiles. By contrast, in lower transmission settings, the effect of seroreversion following first exposure—and the less frequent boosting effect of subsequent exposures—may be more evident in seroprevalence profiles, possibly resulting in a decline in seropositivity in older age groups.

Exposure to *T. solium* is known to be greater for individuals with lower educational levels, those from lower socioeconomic strata [6, 32] and those facing social marginalisation [9, 33–35]. Our findings are consistent with these previously reported findings, with the odds of displaced people testing positive being almost twofold higher than people in the highest socioeconomic stratum. Internal displacement in Colombia is a major issue that often involves the poorest and most disadvantaged people [36], but if the control of *T. solium* is to become comprehensive, displaced people may require enhanced interventions. Health education could be one such option for control in specific populations using tools such as “The Vicious Worm” [37], as there is some evidence that health education campaigns specific to *T. solium* can impact transmission [38]. It is, however, likely that to achieve substantial, sustained reductions in the prevalence of *T. solium* or elimination, particularly in highly endemic areas, a One Health approach targeting the whole *T. solium* system, including infections in pigs, humans and the environment, will be required [39, 40], as recently shown by intervention trials in Peru and Zambia [41, 42].

The only variable related to food and water sources or hygiene practices that was significantly associated with seropositivity to *T. solium* cysticercus antibodies was the use of rainwater. Individuals in households using rainwater as opposed to water stored in wells or cisterns had a 1.6-fold higher odds of seropositivity. Waterborne cysticercosis transmission is supported in the literature, given that the eggs can survive in fresh, brackish and salt waters [32, 43–45] and can contaminate vegetables [45]. Other variables, such as open-field defecation or the use of unsanitary latrines [46, 47], that one might also expect to be associated with exposure to *T. solium* were not identified in our analysis as significant risk factors. We also found that the odds of seropositivity significantly decreased when individuals consumed partially cooked/raw pork meat once per week, an observation possibly confounded by wealth (i.e., wealthier individuals consuming more meat). One might expect that consumption of partially cooked/raw pork meat would be associated with increased odds of seropositivity, given that taeniasis (adult tapeworm) carriers are at risk of autoinfection. However, more research is needed to understand the relative contribution of this route of transmission to overall cysticercosis risk [48].

A particularly striking finding of our analysis was the association between owning dogs and significantly increased odds of test positivity. Dogs in Asia have been reported to test positive for *T. solium* antibodies [49, 50], potentially implicating them as alternative intermediate hosts. Transmission to humans has also been suggested to occur via the consumption of raw or uncooked canine meat [51], although this practice is thought to be extremely rare and not widely reported in Latin America. Moreover, the role of dogs as potential hosts for *T. solium* remains somewhat speculative. Given the coprophagic habits of dogs and their close interaction with humans, it is also possible (and perhaps more likely) that dogs act as mechanical vectors of *T. solium* eggs.

A further striking finding is that among the 10.8% (*n* = 3,154) of individuals owning pigs, we did not find a significantly increased odds of seropositivity, only a non-significant increase in those owning fewer than 10 pigs (possibly indicative of smallholder, subsistence farmers, compared to individuals owning > 10 pigs, which may represent wealthier farmers). A further sub-analysis of pig owners (Additional file 1: Text S2) found no association between seropositivity and pig management practices (e.g. free roaming, feeding wastes, drinking free water, among others). These findings contrast with those reported in other studies in Latin America and other geographical settings, in which human cysticercosis has been associated with owning pigs [2, 33, 52]. Some farming practices, such as using waste or water and mix concentrate as feed, and the lack of drainage systems were non-significantly associated with increased seropositivity. However, because this sub-analysis was based on a much smaller sample (*n* = 3154) with only 388 seropositive individuals, there was limited power to detect significant associations.

In addition to exploring individual and household risk factors associated with exposure to *T. solium*, our geostatistical approach enabled identification of spatial clusters where seropositivity was higher, so-called hotspots (in the north and south of Colombia), or lower (in the central and western areas of the country) than could be explained by the included covariates (Fig. 2). Hotspots where seropositivity was higher than could be explained by the covariates coincided with areas with higher seroprevalence (16‒40%) in the northern coastal area and areas bordering Venezuela (Departments of Atlántico, Magdalena, Cesar, La Guajira), in the northern-central region (Departments of Antioquia and Bolívar), in Vaupés (south-east, bordering Brazil) and in the south, in regions bordering Peru and Brazil (Department of Amazonas; Fig. 1). Neither human nor pig population density was explicitly included in the model and, therefore, these variables could help to explain some of this clustering (because of the potential for increased contamination of the environment with *T. solium* eggs where humans and pigs are abundant). While population densities are heterogeneous across Colombia, some of the highest human population densities are generally found in the north and north-east of the country [53], alongside the highest pig population in the Pacific (east costal), Andean (north-east/north-west) and Caribbean regions (north), as estimated from the Gridded Livestock Database in 2007 [54]. Furthermore, it should be noted that given the level of spatial analysis, we were only able to detect spatial variation at the municipality level.

Local climatic, environmental and ecological conditions may also play a role in the observed clustering. In a recent systematic review, Jansen et al. [45] identified that *Taenia spp*. eggs can survive in the environment for up to 1 year in favourable conditions of high humidity, moderate temperatures (5‒25 °C) and presence of surface water. Moreover, invertebrates, including dung beetles (*Ammophorus rubripes*), can also act as mechanical vectors for the dispersal of *Taenia spp* eggs [55, 56]. Hence, it is highly likely that local conditions—unaccounted for in our statistical model—will influence spatial patterns of exposure.

Although the serosurvey data analysed here are unique in presenting a picture of exposure to *T. solium* cysticercosis at a national scale, geographical coverage is incomplete and the sampling approach may have introduced some biases. In particular, the selection of municipalities with > 5000 individuals and a health centre is likely to have created a bias towards sampling in more densely populated urban areas. This led to an underrepresentation of rural communities, which may typically have had less access to health care and possibly lower overall health. In addition, nine departments were excluded from sampling (due to logistical and resource constraints) and overall, only a relatively small fraction (12%) of Colombia’s municipalities were sampled (133/1122). Women are highly represented, and this is likely due to the decision of randomizing only the individuals present at the interview for inclusion in the study. Also, the data were collected in 2008–2010, over a decade ago, and may therefore not reflect precisely contemporary epidemiological conditions. Nonetheless, we believe that, in the absence of wide-spread national control efforts, the distribution and endemic situation of *T. solium* are unlikely to have changed substantively over the past decade and, therefore, the data provide a useful snapshot of endemic conditions across the country. Due to the nature of surveys, other forms of bias and reverse causation are also possible.

Moreover, it cannot be excluded that any of the encountered associations are confounded by unmeasurable or unknown risk factors and that the a priori decision to drop a certain number of variables might have increased the model residuals, not including possible confounders. On the other hand, the unstructured nature of some variables or the probable collinearity with other exposures made this choice desirable. Despite the lack of data concerning some geographical areas in Colombia, the authors still consider the study outcomes as valuable and indicative of the situation of cysticercosis in the country. In addition, the information provided in the current study could be further used to build models that can spatially predict the disease seroprevalence in non-sampled areas [17], offering a cost-effective tool for decision-makers in places where direct sampling did not take place.

Mapping the distribution and seroprevalence of *T. solium* in endemic countries is a crucial next step in realising the WHO’s goals of implementing intensified control in hyperendemic areas of 17 countries by 2030 [22]. Currently, country-wide data on the transmission of *T. solium*, such as those analysed here for Colombia, are scarce, and thus there is a great deal of work to be done to identify hyperendemic areas in which to implement intensified interventions. Moreover, although working definitions of ‘hyperendemicity’ have been proposed [57], there is not yet a consensus on the definition of endemicity levels for *T. solium* infection. Geostatistical approaches will play an important role in identifying areas of high transmission, particularly if they can be parameterized to identify likely areas of high transmission using Geographical Information System (GIS) data that have comprehensive global coverage. Although our study focused on the identification of risk factors associated with exposure to *T. solium* and residual degrees of spatial clustering, similar geostatistical and machine learning approaches can be used that focus on predicting the spatial distribution of disease using GIS data [17]. Such approaches, conducted at national and global scales, will be crucial in assisting progress towards the WHO’s 2030 goals [22, 58].

## Conclusions

Taeniasis/cysticercosis is a major public health problem and an important cause of epilepsy and other neurological sequelae in many regions of the world. The WHO aims to target this zoonotic NTD with enhanced control where transmission is most intense, although epidemiological data at national and subnational scales remain scarce. The 2008–2010 baseline epidemiological survey undertaken by the Colombian government remains unprecedented in scale and geographical coverage, generating data that are unique and provide a highly valuable resource for understanding the spatial epidemiology of *T. solium* cysticercosis. By taking a contemporary geostatistical approach, we have highlighted key associations between human cysticercosis antibody seropositivity and individual- and household-level risk factors, while also identifying spatial hotspots of exposure, unexplained by the measured covariates. These findings could be used to inform the design of intervention strategies in Colombia, such as targeting spatial hotspots and more highly exposed groups (such as displaced people and women), and also to illustrate how important geostatistical modelling will be as a tool to inform and support the WHO NTD roadmap in its 2021–2030 goals for taeniasis/cysticercosis.

## Supplementary information

**Additional file 1: Table S1.** Notation of parameters used for model building (analysis of residual spatial correlation) and incorporation of spatial structure.** Table S2.** Seroprevalence of* Taenia solium* cysticercus antibodies in Colombia.** Table S3.** Distribution of seropositive individuals, crude odds ratios (ORs) of testing positive for* Taenia solium *cysticercus antibodies by ELISA and associated 95% confidence intervals (CIs) from the univariate mixed-effects model.** Table S4.** Distribution of seropositive individuals, multivariable mixed-effects logistic regression adjusted ORs of testing positive for* Taenia solium* cysticercus antibodies by ELISA and associated CIs.** Table S5.** Pig management sub-set analysis (*n* = 3154). Pig management practices, distribution of seropositive individuals, crude odds ratios (ORs) of testing positive for* Taenia solium* cysticercus antibodies by ELISA and associated 95% CIs. **Figure S1.** Map displaying the sampled municipalities in Colombia (2008–2010). **Text S1.** Supplementary methods. **Text S2.** Results: pig management sub-analysis

## Data Availability

The data that support the findings of this study are available from the Instituto Nacional de Salud (Bogotá, Colombia) but restrictions apply to the availability of these data, which were used under licence for the current study, and so are not publicly available. Data are however available from the authors upon reasonable request and with permission of Instituto Nacional de Salud.
