# Supplementary material for: Spatial distribution and risk factors for human cysticercosis in Colombia
Source: Parasit Vectors. 2021 Nov 27;14:590. doi: 10.1186/s13071-021-05092-8 (PMC8626945; doi:10.1186/s13071-021-05092-8)
Supplement: Supplementary file 1 — Additional file 1: Table S1. Notation of parameters used for model building (analysis of residual spatial correlation) and incorporation of spatial structure. Table S2. Seroprevalence of Taenia solium cysticercus antibodies in Colombia. Table S3. Distribution of seropositive individuals, crude odds ratios (ORs) of testing positive for Taenia solium cysticercus antibodies by ELISA and associated 95% confidence intervals (CIs) from the univariate mixed-effects model. Table S4. Distribution of seropositive individuals, multivariable mixed-effects logistic regression adjusted ORs of testing positive for Taenia solium cysticercus antibodies by ELISA and associated CIs. Table S5. Pig management sub-set analysis (n = 3154). Pig management practices, distribution of seropositive individuals, crude odds ratios (ORs) of testing positive for Taenia solium cysticercus antibodies by ELISA and associated 95% CIs. Figure S1. Map displaying the sampled municipalities in Colombia (2008–2010). Text S1. Supplementary methods. Text S2. Results: pig management sub-analysis [file 13071_2021_5092_MOESM1_ESM.docx]

**Spatial distribution and risk factors for human cysticercosis in Colombia**

Erika Galipó^1,2^, Matthew A. Dixon^3,4,5^, Claudio Fronterrè^6^, Zulma M. Cucunubá^3,4,§^, Maria-Gloria Basáñez^3,4^, Kim Stevens^2^, Astrid Carolina Flórez Sánchez^7^, Martin Walker^2,3^

^1^ Department of Epidemiological Sciences, Animal and Plant Health Agency, New Haw, Addlestone, Surrey, UK

^2^ Department of Pathobiology and Population Sciences and London Centre for Neglected Tropical Disease Research, Royal Veterinary College, Hatfield, UK

^3^ Department of Infectious Disease Epidemiology and London Centre for Neglected Tropical Disease Research, School of Public Health, Imperial College London, London, UK

^4^ Medical Research Centre for Global Infectious Disease Analysis, School of Public Health, Imperial College London, London, UK

^5^ Schistosomiasis Control Initiative (SCI) Foundation, London, UK

^6^ Centre for Health Informatics, Computing and Statistics, Lancaster University, Lancaster, UK

^7^ Grupo de Parasitología, Instituto Nacional de Salud, Bogotá, Colombia

^§^ Present address: Departamento de Epidemiología Clínica, Pontificia Universidad Javeriana, Bogotá, Colombia.

Corresponding author: Matthew A. Dixon: SCI Foundation, Edinburgh House, 170 Kennington Lane, Lambeth, London SE11 5DP, UK. E-mail: [m.dixon15@imperial.ac.uk](mailto:m.dixon15@imperial.ac.uk)

**Additional File: Supplementary Figures, Methods, and Tables**


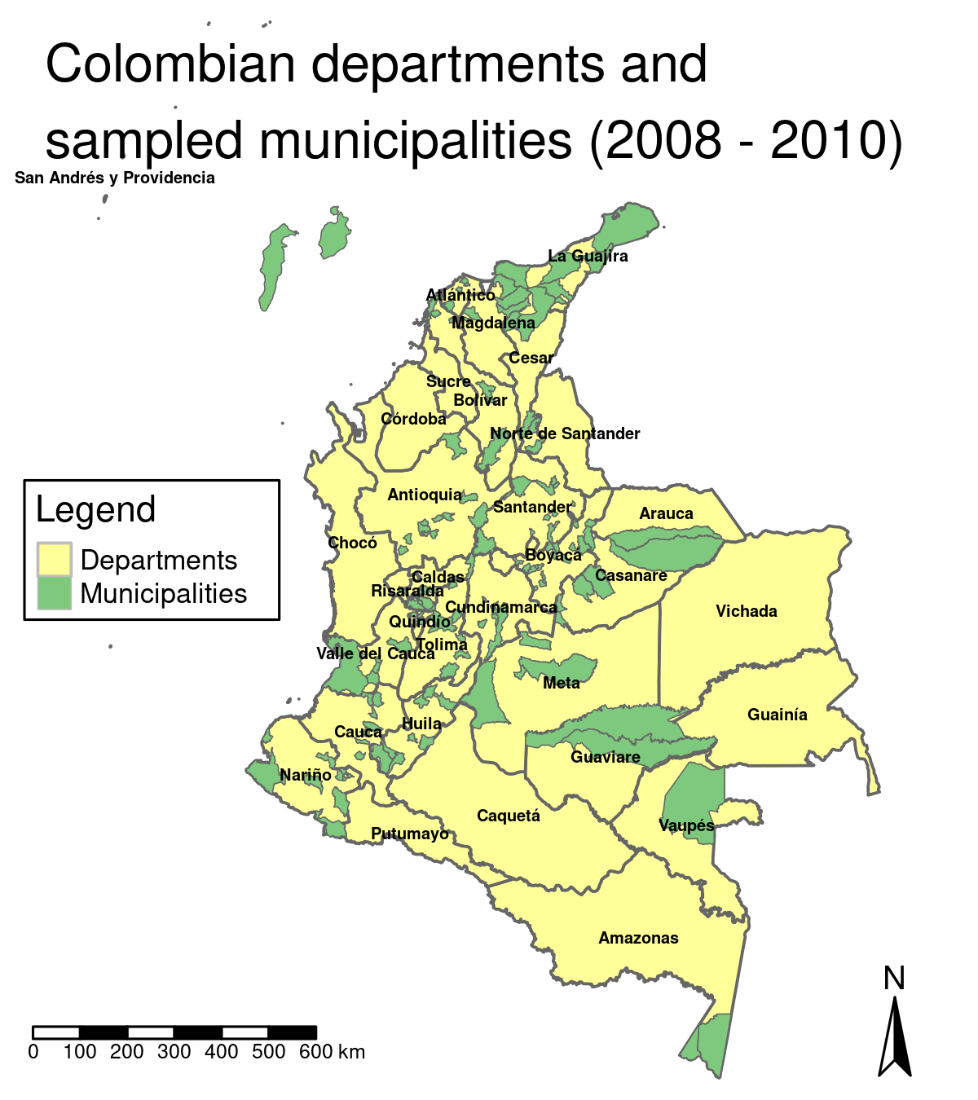


**Figure S1. Map displaying the sampled municipalities in Colombia (2008-2010)**. A total of 133 municipalities were selected for this study. Departments are shown in pale yellow and sampled municipalities in green. Department names are also displayed.

Table S1 presents the notation of parameters used for model building (analysis of residual spatial correlation) and incorporation of spatial structure. Table S2 summarises the seroprevalence of *Taenia solium* cysticercus antibodies in Colombia by department.

**Text S1**

**Supplementary Methods**

**Data cleaning and analysis**

Individual-level seropositivity to *Taenia solium* cysticercus antibodies was defined as the dependent variable in a statistical model to assess association with potential risk factors. Data were cleaned and analysed in R (version 4.0.5) [1]. Age was treated as a categorical variable to allow the risk of infection associated with different age groups to vary in a non-linear manner. Observations with missing values were dropped from the dataset. Geographical clustering variables at area/administrative level were included for each observation in the analysis. In total, 19 explanatory variables were included in the analysis (Table S3), including risk factors relating to sociodemographic information as classified by the DANE [2,3], eating habits, hygienic practices and owning (specific) animals.

**Pig management sub-analysis**

Separate univariate hierarchical models containing a restricted number of observations (*n*=3,154) belonging exclusively to pig owners were explored to evaluate the impact of pig management on seropositivity to *T. solium* cysticercus antibodies. From the final models, adjusted odds ratios (ORs), 95% confidence intervals (95% CI) and *P*-values were obtained for each risk factor. Covariates that returned a *P-*value ≤0.25 were retained for inclusion in a proposed multivariate hierarchical model.

| **Table S1.** Notation of parameters used for model building (analysis of residual spatial correlation) and incorporation of spatial structure. | |
| --- | --- |
| **Parameter** | **Description** |
| $\boldsymbol{Y}$ | Binary vector of observations indicating whether an individual tested positive for *Taenia solium* cysticercus antibodies, assuming a Bernoulli distribution $\mathrm{Bern}\left( \boldsymbol{\mu} \right)$ with $\boldsymbol{\mu}$ a vector of probabilities for testing positive |
| $\boldsymbol{\beta}$ | Vector of regression coefficients |
| $\mathbf{X}$ | Design matrix of explanatory variables |
| $\boldsymbol{U}$ | Vector of independent and normally distributed random effects, $N\left( 0,\tau\right)$, with $\tau$the standard deviation of the municipality random effect terms (indicative of the degree of variability at this level) |
| $\boldsymbol{Z}$ | Vector of independent and normally distributed random effects $N\left( 0,\sigma\right)$, with $\sigma$the standard deviation of the neighbourhood/villages random effect terms (indicative of the degree of variability at this level) |
| $\hat{\boldsymbol{U}}$ | The estimated random effects at the municipality level |
| $\boldsymbol{S(x)}$ | Spatially-structured random effects, where $\boldsymbol{x}$ is a vector with the centroids of the sampled municipalities and $\boldsymbol{S(x)}$ is a spatial Gaussian process with variance $\sigma^{2}$ and correlation function $\rho\left( \mu\right)=\exp\left( - \frac{\mu}{\varphi} \right)$where $\mu$ is the distance between a pair of municipality centroids and $\varphi$ is a parameter that controls the rate at which the spatial correlation decays with increasing distance. |

**Table S2.** Seroprevalence of *Taenia solium* cysticercus antibodies in Colombia

| Department | Number of seropositive participants, *n* | Number of participants, *N* | Seroprevalence (%) | 95% confidence interval |
| --- | --- | --- | --- | --- |
| Vaupés | 441 | 1140 | 38.68 | 35.85 – 41.58 |
| Bolívar | 408 | 1260 | 32.38 | 29.80 – 35.08 |
| Amazonas | 263 | 1210 | 21.74 | 19.44 – 24.17 |
| Cundinamarca | 128 | 891 | 14.37 | 12.13 – 16.84 |
| La Guajira | 173 | 1270 | 13.62 | 11.78 – 15.63 |
| San Andrés | 152 | 1230 | 12.36 | 10.57 – 14.33 |
| Antioquia | 155 | 1291 | 12.01 | 10.28 – 13.90 |
| Cesar | 151 | 1270 | 11.89 | 10.16 – 13.80 |
| Cauca | 142 | 1270 | 11.18 | 9.50 – 13.04 |
| Magdalena | 124 | 1260 | 9.84 | 8.25 – 11.62 |
| Atlántico | 116 | 1280 | 9.06 | 7.55 – 10.77 |
| Nariño | 80 | 1264 | 6.33 | 5.05 – 7.82 |
| Valle del Cauca | 62 | 1260 | 4.92 | 3.79 – 6.26 |
| Tolima | 59 | 1270 | 4.65 | 3.56 – 5.95 |
| Meta | 55 | 1262 | 4.36 | 3.30 – 5.64 |
| Boyacá | 51 | 1270 | 4.02 | 3.00 – 5.25 |
| Bogotá D.C. | 30 | 850 | 3.53 | 2.39 – 5.00 |
| Huila | 44 | 1280 | 3.44 | 2.51 – 4.59 |
| Casanare | 35 | 1252 | 2.80 | 1.95 – 3.87 |
| Guaviare | 33 | 1220 | 2.70 | 1.87 – 3.78 |
| Santander | 32 | 1270 | 2.52 | 1.73 – 3.54 |
| Quindio | 28 | 1260 | 2.22 | 1.48 – 3.20 |
| Risaralda | 17 | 1270 | 1.34 | 0.78 – 2.13 |
| Caldas | 6 | 1260 | 0.48 | 0.17 – 1.03 |

**Table S3**. Distribution of seropositive individuals, crude odds ratios (ORs) of testing positive for *Taenia solium* cysticercus antibodies by ELISA and associated 95% confidence intervals (CIs) from the univariate mixed-effects model*.*

|  | | **Total positive (%; 95% CI)** | **CrudeORs** | **95% CI** | | ***P*-value*** | |
| --- | --- | --- | --- | --- | --- | --- | --- |
| **Sociodemographic characteristics** | | | | | | | |
| **Sex** | Male | 809 (8.77; 8.20 – 9.36) | 1 |  | **<0.0001** | |  |
|  | Female | 1967 (9.82; 9.41 – 10.20) | 1.31 | 1.19 – 1.44 |  |  |  |
| **Age groups (years)** | 2–10 | 76 (7.82; 6.21 – 9.69) | 1 |  | **0.0027** | |  |
|  | 11–20 | 337 (9.41; 8.47 – 10.40) | 1.50 | 1.13 – 1.99 |  |  |  |
|  | 21–30 | 641 (9.78; 9.07 – 10.50) | 1.54 | 1.17 – 2.02 |  |  |  |
|  | 31–40 | 617 (9.70; 8.99 – 10.50) | 1.61 | 1.23 – 2.11 |  |  |  |
|  | 41–50 | 578 (9.76; 9.01 – 10.50) | 1.76 | 1.34 – 2.31 |  |  |  |
|  | 51–60 | 409 (9.30; 8.46 – 10.20) | 1.73 | 1.31 – 2.29 |  |  |  |
|  | 61–64 | 117 (8.04; 6.70 – 9.56) | 1.49 | 1.08 – 2.07 |  |  |  |
| **Residence** | Rural | 764 (11.80; 11.00 – 12.60) | 1 |  | **<0.0001** | |  |
|  | Urban | 2011 (8.83; 8.46 – 9.20) | 0.61 | 0.53 – 0.71 |  |  |  |
| **Education level** | Education higher than secondary | 296 (7.67; 6.85 – 8.55) | 1 |  | **<0.0001** | |  |
|  | Partial/ complete secondary | 1190 (8.88; 8.40 – 9.37) | 1.12 | 0.97 – 1.29 |  |  |  |
|  | Partial/complete primary | 1113 (10.5; 9.91 – 11.10) | 1.43 | 1.24 – 1.66 |  |  |  |
|  | No education | 176 (12.8; 11.10 – 14.70) | 1.45 | 1.16 – 1.82 |  |  |  |
| **Occupation** | Other occupations | 125 (6.93; 5.80 – 8.20) | 1 |  | **0.00014** | |  |
|  | Self-employed | 325 (7.49; 6.72 – 8.31) | 1.02 | 0.81 – 1.23 |  |  |  |
|  | Employee | 374 (8.56; 7.75 – 9.43) | 1.06 | 0.85 – 1.33 |  |  |  |
|  | Farm coordinator | 12 (8.89; 4.68 – 15.00) | 1.19 | 0.60– 2.35 |  |  |  |
|  | Farm labourer | 65 (9.03; 7.04 – 11.40) | 1.22 | 0.87 – 1.73 |  |  |  |
|  | Student | 322 (9.10; 8.18 – 10.10) | 1.06 | 0.84 – 1.33 |  |  |  |
|  | Housewife/houseman | 1313 (10.1; 9.58 – 10.6) | 1.33 | 1.08 – 1.62 |  |  |  |
|  | Businessman | 53 (10.6; 8.06 – 13.7) | 1.27 | 0.87 – 1.83 |  |  |  |
|  | Farm owner | 186 (22.00; 19.30 – 25.00) | 1.56 | 1.17 – 2.08 |  |  |  |
| **Socio-economic status** | ≥4 | 26 (4.48; 2.94 – 6.49) | 1 |  | **<0.0001** | |  |
|  | 3 | 176 (4.70; 4.05 – 5.43) | 0.79 | 0.49 – 1.27 |  |  |  |
|  | 2 | 706 (6.95; 6.46 – 7.46) | 0.91 | 0.57 – 1.44 |  |  |  |
|  | 1 | 1715 (11.90; 11.30 – 12.40) | 1.23 | 0.78 – 1.95 |  |  |  |
|  | Displaced people | 152 (51.00; 45.20 – 56.80) | 3.06 | 1.65 – 5.66 |  |  |  |
| **Eating habits** | | | | | | | |
| **Pork con-sumption & cooking level** | No consumption | 416 (12.50; 11.40 – 13.70) | 1 |  | 0.12 | |  |
|  | Well cooked; <once per month | 1203 (9.44; 8.94 – 9.96) | 0.89 | 0.78 – 1.02 |  |  |  |
|  | Well cooked; once per month | 561 (9.69; 8.94 – 10.5) | 0.89 | 0.76 – 1.04 |  |  |  |
|  | Well cooked; once per week | 293 (8.57; 7.65 – 9.56) | 0.78 | 0.66 – 0.94 |  |  |  |
|  | Well cooked; >once per week | 127 (8.20; 6.88 – 9.68) | 0.88 | 0.70 – 1.11 |  |  |  |
|  | Partially cooked/raw; <once per month | 96 (7.27; 5.93 – 8.81) | 0.79 | 0.61 – 1.02 |  |  |  |
|  | Partially cooked/raw; once per month | 32 (7.34; 5.07 – 10.2) | 0.82 | 0.55 – 1.21 |  |  |  |
|  | Partially cooked/raw; once per week | 26 (6.33; 4.17 – 9.13) | 0.54 | 0.35 – 0.84 |  |  |  |
|  | Partially cooked/raw; >once per week | 21 (7.92; 4.97 – 11.9) | 0.82 | 0.50 – 1.33 |  |  |  |
| **Food con-sumption in streets** | Never consumed food in street | 533 (9.58; 8.82 – 10.4) | 1 |  | 0.42 | |  |
|  | Consumed food in street | 2242 (9.47; 9.10 – 9.85) | 0.95 | 0.85 – 1.07 |  |  |  |
| **Water source** | Well/ cistern | 190 (6.94; 6.02 – 7.96) | 1 |  | **<0.0001** | |  |
|  | Aqueduct | 1401 (7.19; 6.83 – 7.56) | 0.89 | 0.72 – 1.10 |  |  |  |
|  | Waterway | 564 (11.50; 10.60 – 12.5) | 1.24 | 0.98 – 1.57 |  |  |  |
|  | Other sources | 132 (23.00; 19.60 – 26.70) | 1.21 | 0.86 – 1.71 |  |  |  |
|  | Rain water | 488 (31.40; 29.10 – 33.70) | 1.95 | 1.49 – 2.55 |  |  |  |
| **Hygiene practices** | | | | | | | |
| **Washing vegetables** | No consumption | 8 (9.76; 4.31 – 18.30) | 1 |  | **0.05** | |  |
|  | Always washed | 588 (8.05; 7.43 – 8.69) | 1.17 | 0.53 – 2.58 |  |  |  |
|  | Occasionally washed | 1461 (13.7; 13.00 – 14.30) | 1.34 | 0.61 – 2.95 |  |  |  |
|  | Never washed | 718 (6.43; 5.98 – 6.90) | 1.09 | 0.49 – 2.41 |  |  |  |
| **Washing hands after toilette usage** | Always | 1549 (8.69; 8.28 – 9.11) | 1 |  | 0.28 | |  |
|  | Occasionally washed | 1144 (10.80; 10.20 – 11.40) | 1.07 | 0.97 – 1.17 |  |  |  |
|  | Never washed | 82 (9.82; 7.89 – 12.00) | 0.91 | 0.70 – 1.18 |  |  |  |
| **Washing hands before a meal** | Always washed | 1171 (9.06; 8.58 – 9.57) | 1 |  | 0.24 | |  |
|  | Occasionally washed | 1489 (9.96; 9.48 – 10.40) | 1.04 | 0.95 – 1.14 |  |  |  |
|  | Never washed | 115 (8.36; 6.95 – 9.95) | 0.87 | 0.70 – 1.08 |  |  |  |
| **Elimination of excreta** | Sanitary | 1526 (7.48; 7.12 – 7.85) | 1 |  | **<0.0001** | |  |
|  | In waterway | 50 (9.21; 6.91 – 12.00) | 1.12 | 0.75 – 1.69 |  |  |  |
|  | Latrine with well | 862 (12.9; 12.1 – 13.7) | 1.48 | 1.29 – 1.70 |  |  |  |
|  | Latrine without well | 67 (12.9; 10.1 – 16.1) | 1.61 | 1.16 – 2.21 |  |  |  |
|  | Open field | 270 (24.9; 22.3 – 27.5) | 1.76 | 1.41 – 2.19 |  |  |  |
| **Owning animals** | | | | | | | |
| **Owning cattle** | Not owning | 2641 (9.45; 9.11 – 9.80) | 1 |  | 0.16 | |  |
|  | Owning | 134 (10.3; 8.74 – 12.1) | 1.18 | 0.95 – 1.47 |  |  |  |
| **Owning cats** | Not owning | 2208 (9.36; 8.99 – 9.74) | 1 |  | **0.01** | |  |
|  | Owning | 567 (10.0; 9.26 – 10.8) | 1.16 | 1.04 – 1.30 |  |  |  |
| **Owing dogs** | Not owning | 1349 (8.33; 7.91 – 8.77) | 1 |  | **<0.0001** | |  |
|  | Owning | 1426 (10.9; 10.4 – 11.5) | 1.26 | 1.15 – 1.37 |  |  |  |
| **Owing birds** | Not owning | 1799 (8.90; 8.51 – 9.30) | 1 |  | **0.04** | |  |
|  | Owning | 976 (10.80; 10.20 – 11.50) | 1.11 | 1.01 – 1.23 |  |  |  |
| **Owning pigs** | Not owning | 2386 (9.14; 8.80 – 9.50) | 1 |  | **0.0053** | |  |
|  | Owning ≤10 pigs | 349 (12.9; 11.70 – 14.30) | 1.31 | 1.12 – 1.53 |  |  |  |
|  | Owning >10 pigs | 40 (8.70; 6.28 – 11.7) | 0.93 | 0.65 – 1.32 |  |  |  |
| **Owing other animals** | Not owning | 2613 (9.45; 9.11 – 9.80) | 1 |  | 0.58 | |  |
|  | Owning | 162 (10.1; 8.69 – 11.7) | 1.06 | 0.87 – 1.28 |  |  |  |

** P*-values obtained from significance testing (of fixed-effects) using a likelihood ratio test.

**Table S4.** Distribution of seropositive individuals, multivariable mixed-effects logistic regression adjusted odds ratios (ORs) of testing positive for *Taenia solium* cysticercus antibodies by ELISA and associated confidence intervals (CIs)*.*

|  | | **Total positive (%; 95% CI)** | **Adj ORs** | **95% CI** | ***P*-value*** |
| --- | --- | --- | --- | --- | --- |
| **Sociodemographic characteristics** | | | | | |
| **Sex** | Male | 809 (8.77; 8.20 – 9.36) | 1 |  | **<0.0001** |
|  | Female | 1967 (9.82; 9.41 – 10.20) | 1.29 | 1.15 – 1.45 |  |
| **Age groups**  **(years)** | 2–10 | 76 (7.82; 6.21 – 9.69) | 1 |  | **0.0007** |
|  | 11–20 | 337 (9.41; 8.47 – 10.4) | 1.83 | 1.35 – 2.46 |  |
|  | 21–30 | 641 (9.78; 9.07 – 10.50) | 1.95 | 1.43 – 2.67 |  |
|  | 31–40 | 617 (9.70; 8.99 – 10.50) | 1.95 | 1.42 – 2.68 |  |
|  | 41–50 | 578 (9.76; 9.01 – 10.50) | 2.10 | 1.53 – 2.89 |  |
|  | 51–60 | 409 (9.30; 8.46 – 10.20) | 2.00 | 1.45 – 2.75 |  |
|  | 61–64 | 117 (8.04; 6.70 – 9.56) | 1.67 | 1.16 – 2.40 |  |
| **Residence** | Rural | 764 (11.80; 11.00 – 12.60) | 1 |  | 0.21 |
|  | Urban | 2011 (8.83; 8.46 – 9.20) | 0.88 | 0.73 – 1.07 |  |
| **Education level** | Education higher than secondary | 296 (7.67; 6.85 – 8.55) | 1 |  | **0.0002** |
|  | Partial/ complete secondary | 1190 (8.88; 8.40 – 9.37) | 1.05 | 0.90 – 1.21 |  |
|  | Partial/complete primary | 1113 (10.5; 9.91 – 11.10) | 1.31 | 1.11 – 1.54 |  |
|  | No education | 176 (12.8; 11.10 – 14.70) | 1.35 | 1.05 – 1.72 |  |
| **Occupation** | Other occupations | 125 (6.93; 5.80 – 8.20) | 1 |  | 0.80 |
|  | Self-employed | 325 (7.49; 6.72 – 8.31) | 1.01 | 0.81 – 1.27 |  |
|  | Employee | 374 (8.56; 7.75 – 9.43) | 1.08 | 0.85 – 1.36 |  |
|  | Farm coordinator | 12 (8.89; 4.68 – 15.00) | 1.05 | 0.53– 2.06 |  |
|  | Farm labourer | 65 (9.03; 7.04 – 11.40) | 1.06 | 0.75 – 1.51 |  |
|  | Student | 322 (9.10; 8.18 – 10.10) | 1.20 | 0.92 – 1.56 |  |
|  | Housewife/houseman | 1313 (10.1; 9.58 – 10.6) | 1.07 | 0.87 – 1.33 |  |
|  | Businessman | 53 (10.6; 8.06 – 13.7) | 1.23 | 0.84 – 1.79 |  |
|  | Farm owner | 186 (22.00; 19.30 – 25.00) | 1.21 | 0.90 – 1.63 |  |
| **Socio-economic status** | ≥4 | 26 (4.48; 2.94 – 6.49) | 1 |  | **0.008** |
|  | 3 | 176 (4.70; 4.05 – 5.43) | 0.82 | 0.51 – 1.33 |  |
|  | 2 | 706 (6.95; 6.46 – 7.46) | 0.89 | 0.56 – 1.41 |  |
|  | 1 | 1715 (11.90; 11.30 – 12.40) | 1.01 | 0.63 – 1.62 |  |
|  | Displaced people | 152 (51.00; 45.20 – 56.80) | 1.94 | 1.02 – 3.70 |  |
| **Eating habits** | | | | | |
| **Pork con-sumption & cooking level** | No consumption | 416 (12.50; 11.40 – 13.70) | 1 |  | 0.37 |
|  | Well cooked; <once per month | 1203 (9.44; 8.94 – 9.96) | 0.92 | 0.80 – 1.05 |  |
|  | Well cooked; once per month | 561 (9.69; 8.94 – 10.5) | 0.94 | 0.80 – 1.11 |  |
|  | Well cooked; once per week | 293 (8.57; 7.65 – 9.56) | 0.85 | 0.71 – 1.02 |  |
|  | Well cooked; >once per week | 127 (8.20; 6.88 – 9.68) | 0.97 | 0.77 – 1.23 |  |
|  | Partially cooked/raw; <once per month | 96 (7.27; 5.93 – 8.81) | 0.84 | 0.65 – 1.09 |  |
|  | Partially cooked/raw; once per month | 32 (7.34; 5.07 – 10.2) | 0.89 | 0.59 – 1.32 |  |
|  | Partially cooked/raw; once per week | 26 (6.33; 4.17 – 9.13) | 0.58 | 0.38 – 0.90 |  |
|  | Partially cooked/raw; >once per week | 21 (7.92; 4.97 – 11.9) | 0.93 | 0.57 – 1.52 |  |
| **Water source** | Well/ cistern | 190 (6.94; 6.02 – 7.96) | 1 |  | **0.004** |
|  | Aqueduct | 1401 (7.19; 6.83 – 7.56) | 1.05 | 0.84 – 1.31 |  |
|  | Waterway | 564 (11.50; 10.60 – 12.5) | 1.16 | 0.91 – 1.48 |  |
|  | Other sources | 132 (23.00; 19.60 – 26.70) | 1.13 | 0.80 – 1.60 |  |
|  | Rain water | 488 (31.40; 29.10 – 33.70) | 1.62 | 1.23 – 2.14 |  |
| **Hygiene practices** | | | | | |
| **Washing vegetables** | No consumption | 8 (9.76; 4.31 – 18.30) | 1 |  | 0.51 |
|  | Always washed | 588 (8.05; 7.43 – 8.69) | 1.23 | 0.55 – 2.75 |  |
|  | Occasionally washed | 1461 (13.7; 13.00 – 14.30) | 1.31 | 0.59 – 2.93 |  |
|  | Never washed | 718 (6.43; 5.98 – 6.90) | 1.18 | 0.53 – 2.65 |  |
| **Washing hands before a meal** | Always washed | 1171 (9.06; 8.58 – 9.57) | 1 |  | 0.38 |
|  | Occasionally washed | 1489 (9.96; 9.48 – 10.40) | 1.04 | 0.94 – 1.15 |  |
|  | Never washed | 115 (8.36; 6.95 – 9.95) | 0.90 | 0.72 – 1.13 |  |
| **Elimination of excreta** | Sanitary | 1526 (7.48; 7.12 – 7.85) | 1 |  | 0.42 |
|  | In waterway | 50 (9.21; 6.91 – 12.00) | 0.84 | 0.56 – 1.28 |  |
|  | Latrine with well | 862 (12.9; 12.1 – 13.7) | 1.11 | 0.94 – 1.31 |  |
|  | Latrine without well | 67 (12.9; 10.1 – 16.1) | 1.20 | 0.86 – 1.68 |  |
|  | Open field | 270 (24.9; 22.3 – 27.5) | 1.16 | 0.90 – 1.49 |  |
| **Owning animals** | | | | | |
| **Owning cattle** | Not owning | 2641 (9.45; 9.11 – 9.80) | 1 |  | 0.67 |
|  | Owning | 134 (10.3; 8.74 – 12.1) | 0.95 | 0.75 – 1.20 |  |
| **Owning cats** | Not owning | 2208 (9.36; 8.99 – 9.74) | 1 |  | 0.29 |
|  | Owning | 567 (10.0; 9.26 – 10.8) | 1.06 | 0.94 – 1.20 |  |
| **Owing dogs** | Not owning | 1349 (8.33; 7.91 – 8.77) | 1 |  | **0.0005** |
|  | Owning | 1426 (10.9; 10.4 – 11.5) | 1.18 | 1.08 – 1.30 |  |
| **Owing birds** | Not owning | 1799 (8.90; 8.51 – 9.30) | 1 |  | 0.45 |
|  | Owning | 976 (10.80; 10.20 – 11.50) | 0.96 | 0.86 – 1.07 |  |
| **Owning pigs** | Not owing | 2386 (9.14; 8.80 – 9.50) | 1 |  | 0.19 |
|  | Owning ≤10 pigs | 349 (12.9; 11.70 – 14.30) | 1.13 | 0.96 – 1.33 |  |
|  | Owning >10 pigs | 40 (8.70; 6.28 – 11.7) | 0.85 | 0.59 – 1.22 |  |

** P*-values obtained through Analysis of Deviance; Type II Wald Chi-square test, using car::Anova function in R.

**Text S2.**

**Results**

**Pig management sub-analysis**

Within the variables describing pig management, a total of 3,154 observations were kept after dataset cleaning and manipulation. At the univariate analysis, none of the included pig management risk factors was associated with being positive to *T. solium* cysticercus antibodies and the *P-*values were all above the chosen 0.25 cut off for inclusion in a multivariate model. No further analysis was carried out for these covariates and sub-analysis.

**Table S5. Pig management sub-set analysis (n = 3,154)**. Pig management practices, distribution of seropositive individuals, crude odds ratios (ORs) of testing positive for *Taenia solium* cysticercus antibodies by ELISA and associated 95% confidence intervals (CIs)

|  | | **Total positive (%; 95% CI)** | | | **Crude ORs** | **95% CI** | ***P*-value*** |
| --- | --- | --- | --- | --- | --- | --- | --- |
| **Pigs’ housing** | Pens | | 161 (10.5; 8.99 – 12.1) | 1 | |  | 0.90 |
|  | Free | | 189 (13.8; 12.0 – 15.8) | 0.93 | | 0.69 – 1.26 |  |
|  | Both | | 38 (15.1; 10.9 – 20.2) | 0.95 | | 0.57 – 1.57 |  |
| **Pigs’ feed** | Concentrate | | 31 (9.20; 6.34 – 12.8) | 1 | |  | 0.58 |
|  | Mix concentrate and wastes | | 64 (9.14; 7.11 – 11.5) | 1.17 | | 0.69 – 1.98 |  |
|  | Waste | | 293 (13.8; 12.4 – 15.4) | 1.28 | | 0.79 – 2.07 |  |
| **Pigs’ beverage** | Fountains | | 167 (10.9; 9.38 – 12.6) | 1 | |  | 0.91 |
|  | Free water | | 187 (13.5; 11.8 – 15.4) | 0.94 | | 0.69 – 1.26 |  |
|  | Both | | 34 (14.2; 10.1 – 19.3) | 0.96 | | 0.57 – 1.63 |  |
| **Pigs’ manure elimination** | Drainage system | | 22 (8.24; 5.24 – 12.20) | 1 | |  | 0.93 |
|  | Mix with and without drainage | | 6 (11.30; 4.27 – 23.0) | 1.16 | | 0.37 – 3.62 |  |
|  | Without drainage | | 360 (12.70; 11.50 – 14.0) | 1.10 | | 0.65 – 1.89 |  |

Sub-dataset analysis (only those individuals owning pigs).

** P*-values obtained from significance testing (of fixed-effects) likelihood ratio test*.*

**Supplementary References**

1. R-Core-Team. R: A language and environment for statistical computing. Vienna, Austria: R Foundation for Statistical Computing; 2015. Available: <https://www.r-project.org/>.
2. National Administrative Department of Statistics (DANE). Información técnica. Censo Nacional de Población y Vivienda 2018. 2020. Available from: <https://www.dane.gov.co/index.php/estadisticas-por-tema/demografia-y-poblacion/censo-nacional-de-poblacion-y-vivenda-2018/informacion-tecnica>.
3. National Administrative Department of Statistics (DANE). Social Stratification in the Consumer Price Index (CPI). DANE Colombia 2015. 2016. Available from: <https://unece.org/fileadmin/DAM/stats/documents/ece/ces/ge.22/2016/Session_3_Colombia_socioeconomic_stratification.pdf>.
